# Using the Positive Peers Mobile App to Improve Clinical Outcomes for Young People With HIV: Prospective Observational Cohort Comparison

**DOI:** 10.2196/37868

**Published:** 2022-09-28

**Authors:** Mary M Step, Jennifer McMillen Smith, Steven A Lewis, Ann K Avery

**Affiliations:** 1 College of Public Health Kent State University Kent, OH United States; 2 Division of Social Work Metrohealth System Cleveland, OH United States; 3 Center for Health Care Research and Policy Population Health Research Institute Case Western Reserve University School of Medicine at The MetroHealth System Cleveland, OH United States; 4 Division of Infectious Diseases Case Western Reserve University School of Medicine at The MetroHealth System Cleveland, OH United States

**Keywords:** mobile health, mHealth, HIV, Positive Peers, retention in care, re-engagement in care, viral suppression

## Abstract

**Background:**

Disparities in HIV outcomes persist among racial, gender, and sexual minorities in the United States. Younger people face a greater risk of contracting HIV, often living without knowledge of their HIV status for long periods. The Positive Peers App (PPA) is a multifunctional HIV support tool designed to improve HIV-related clinical outcomes for young people with HIV. The app was designed according to the specifications of an in-care young adult HIV community in Northeast Ohio. Data provided in this study provide preliminary evidence of the usefulness of PPA as a relevant tool for engaging this clinical patient population in care and facilitating viral suppression.

**Objective:**

In this study, we aimed to describe variations in PPA use and examine the associations between use and HIV clinical outcomes between self-selected user and nonuser cohorts in the same clinical population.

**Methods:**

The PPA was offered free of charge to persons with HIV, aged 13 to 34 years of age, diagnosed with HIV within the last 12 months, out of care for 6 months during the last 24 months, or not virally suppressed (HIV viral load >200 copies/mL) in the prior 24 months. Baseline and 6- and 12-month surveys were administered via an audio computer-assisted self-interviewing system to all participants. The app’s user activity was tracked natively by the app and stored in a secure server. Participant demographic and HIV care data were extracted from clinical records within 12 months before the start of the study and across the duration of the study period. HIV care outcomes of PPA users (n=114) were compared with those of nonusers (n=145) at the end of the study period (n=259).

**Results:**

The analysis showed that younger PPA users (aged 13-24 years) were more likely to obtain HIV laboratories (adjusted odds ratio 2.85, 95% CI 1.03-7.90) and achieve sustained viral suppression than nonusers (adjusted odds ratio 4.2, 95% CI 1.2-13.9).

**Conclusions:**

The PPA appears to help younger users sustain HIV suppression. The app offers an important tool for addressing this critical population. The PPA remains in the field and is currently being adopted by other localities to facilitate their efforts to end the HIV epidemic. Although our reported observational results require additional validation and stringent ongoing surveillance, the results represent our best efforts in a pilot study to provide a measure of efficacy for the PPA. Next steps include a large-scale evaluation of the PPA acceptability and effectiveness. Given the building evidence of user reports and outcomes, the freely available PPA could be a helpful tool for achieving Ending the HIV Epidemic goals.

## Introduction

### Background

Although clinical outcomes have improved in adults diagnosed with HIV [1], significant disparities remain for young people [2-4]. Most new HIV diagnoses in the United States are among adolescents and young adults [5]. Although ≥60% of younger (aged 13-24 years) people living with HIV are virally suppressed, significantly fewer Black and Latinx demographic groups are not [4]. Furthermore, among all young people, close to 80% are transgender or cisgender males, who most often (69%) reported HIV transmission as occurring via male-to-male sexual contact [4,5]. Given these trends, young people with HIV can experience the intersection of multiple disenfranchised communities, resulting in compounded stigma, social and family isolation, and socially determined barriers to HIV care [6-8]. The downstream effects of this burden can determine decreased lifetime health and overall longevity. Importantly, tailored mobile health interventions have been shown to effectively reduce HIV disparities for younger people and those who identify with a gender or sexual minority identity [9-12].

Mobile health apps can harness the dissemination dynamics of social media either by linking to existing platforms or by creating networks of people with similar health challenges [13]. As social media networks allow for a more user-centric, collaborative communication process, they offer greater opportunities for engagement with both health information and similar others [13,14]. However, although several studies have shown that social media platforms can serve as an effective channel for disseminating information [15,16], fewer studies link to health outcomes or identify mechanisms for change [17]. Therefore, mobile platforms that offer an effective interface for receiving tailored HIV-related information, track use, and afford users an opportunity to engage in their own recovery may have a meaningful impact on the HIV care cascade.

### Prior Work

The Positive Peers App (PPA) was created as a suite of app functions that can address the range of possible needs a young person living with HIV might have [18]. Following the formation of a community advisory board, we developed specific technical features that promoted user agency to best address users’ needs and provide continuous, vetted, and tailored content directly to demographically defined user groups. The resulting PPA is the center of a social media–supported network that consists of a website, Instagram, TikTok, and Twitter feed that reaches out to the HIV community with a stream of evidence-based HIV-relevant content, targeted at adolescent and young adult user groups. PPA functions range from passive to highly, including the provision of local resources (eg, housing and counseling), topical blogs, narrative accounts, medication reminders, a community forum and private chat. Evidence to date supports the PPA as being acceptable and received as intended by users [18,19]. User feedback suggests that the shared experience found among PPA users is perceived as both restorative and transitional [19]. PPA use has been linked to decreased perceived stigma, and users report that the privacy, opportunities for private instant chat, and simple self-management tools provide a useful, safe, and supportive space protected from discrimination and judgment [19].

Generally, we expect that the more a user engages with personally relevant aspects of the app, the more likely the person is to learn from the app content and form internet-based supportive relationships with other users. This prediction rests on a user-centric model of mobile app use that suggests user’s needs and characteristics of the technology interact to determine user engagement [20-22]. We expect greater engagement with the mobile app to influence the acceptance of promoted HIV messaging and increase the likelihood of desirable HIV clinical outcomes [23].

### Goal of This Study

Although users report liking the PPA and community [19], it is important to evaluate whether the app provides a clinical benefit to users. Our aim for this study was to determine whether PPA use provides a clinical benefit to young people living with HIV. We expect that (1) PPA users will be more engaged in care than a nonparticipating cohort from the same clinic and (2) PPA users will demonstrate greater viral suppression than those who do not use the app. In addition, we aim to learn whether relevant user demographics or personal characteristics are associated with app use or whether defined user engagement groups experience greater or lesser benefits.

## Methods

### Research Design

The parent study for this work was designed to develop, build, and analyze the feasibility and acceptability of the PPA by the targeted user group [18]. This study used a prospective observational single-cohort design, with measures assessed at baseline and at 6, 12, and 18 months. This study was designed after the app was introduced in the field to extend our evaluation of PPA use to HIV clinical outcomes.

App use was logged in real time and tracked directly by the operating system of the app. Clinical outcome data before and after PPA use were obtained from the electronic health record. We recognize that although a randomized controlled trial is ideal for isolating predicted effects, this pilot demonstration project was preceded by a lengthy preliminary design stage that precluded an additional clinical trial evaluation. Consequently, a cohort comparison design of PPA users and eligibility-matched nonusers within the same clinical population during the same time frame provided a reasonable option for evaluating clinical outcomes retrospectively [24] during the study period (October 2016 to May 2019).

### Ethics Approval

This study was approved by the Institutional Review Board for Human Research Protections of the MetroHealth System (IRB:15-00741) on July 20, 2016, and is reviewed annually.

### Participants

The study sample was derived from the HIV clinic population at a public hospital in Cleveland, Ohio, which serves as the primary source of medical care for the surrounding underserved neighborhoods in and around Cuyahoga County. Eligibility requirements for participation included (1) age between 13 and 34 years; (2) receiving HIV care within the health system; and (3) an HIV diagnosis within the last 12 months, out of care for 6 months during the last 24 months, or lack of viral suppression (HIV viral load >200 copies/mL) in the previous 24 months. Essentially, the participants were either newly diagnosed or not fully engaged in HIV care.

### Study Recruitment and Comparison Cohort

Potential participants were first identified via an electronic health record query and referrals from clinic staff. At the end of recruitment, the study sample included 114 young people with HIV who remained enrolled for the duration of the study period.

A local cohort of young people with HIV not enrolled in the PPA pilot was identified for comparison with the pilot sample of PPA users. This comparison cohort (n=259) comprised patients who met the same eligibility criteria for enrollment in the parent PPA study and had a visit to the HIV clinic during the enrollment period but did not enroll to use the app or participate in the study. A manual chart review confirmed the eligibility criteria for the entire sample and provided a record of all clinic visits and laboratory results completed during the study period. It is unknown whether non-PPA user patients were invited and declined study participation or were simply not made aware of the app.

### Measurement

#### Demographic Characteristics

Demographic information was collected from the PPA participants and comparison cohort. Variables included age, race, ethnicity, education, employment status, sexual orientation, and incarceration history, all known social determinants that influence disparities in HIV outcomes [25-29]. Age was categorized as 13 to 24, 25 to 29, and 30 to 34 years to facilitate comparisons among commonly defined age classes in HIV research [26]. Race and ethnicity were reported using the US Census Bureau categories. Finally, respondents were asked to report the number of times they were incarcerated in a jail or prison. Incarceration history was categorized for analysis as none, 1 or 2 times, or ≥3 times.

#### PPA Engagement

PPA engagement among app users was assessed directly from native app performance data associated with each user and stored on a secure server. Variables included the number of times the user logged in and the number of acts the user completed while logged in. Wide variability was observed across these app variables. Consequently, we categorized the number of user acts variable to better compare the user’s app activity. Using the median value as a cutoff point, 3 ordinal categories of PPA use were created based on the number of actions a user took during the first 3 months of having the app on their phone: 0, none; 1, low or moderate (at or below median use); and 2, high (above median use).

#### HIV Outcomes

Consistent with the Health Resources Service Administration Ryan White program standards, HIV outcomes included engagement in care and HIV viral suppression [26]. Engagement in care was coded as *yes* if that had an office visit or laboratory tests completed at both 6 and 12 months for prestudy or poststudy entry. Viral suppression was coded *yes* if the viral load was less than 200 copies per ml at both 6 and 12 months following diagnosis.

#### Statistical Analysis

Baseline characteristics were examined across and within samples of PPA participants and the nonparticipating cohort where Pearson chi-square test was used to formally test for differences between the app user and nonuser groups. Full data were present for all characteristics except race, for which ≤3% of the values were missing.

App user activity, including the number of log-ins, features used, and number of user acts, was examined for PPA participants during the 6-month period following enrollment. Inspection of these data showed that app activity tended to diminish rapidly after 3 months. Consequently, user engagement with the app was derived from app activity in the first 3 months. Medians and IQRs were reported for the number of log-ins and the number of user acts owing to the noted skewness in distributions. Formal tests of significance were not performed because of concerns regarding the sample size.

To test the contribution of app use to outcomes, 3 separate logistic regression models were developed by regressing each HIV outcome (ie, office visits, completion of HIV laboratory tests and HIV viral suppression) on PPA participation (yes or no), while controlling for baseline characteristics and measures. Interaction effects were tested in the models to assess the potential for effect modification relative to PPA participation and individual characteristics. For each outcome modeled, odds ratios and corresponding 95% CIs were reported for PPA participation versus nonparticipation in either the overall or stratified models (in cases of significant interaction or effect modification).

In addition, outcomes were evaluated from the medical records before participation (before using the PPA) and following participation (after using the PPA) to determine if those measures had significantly changed for PPA participants. A McNemar test of agreement was used as a formal test of differences. We also examined the differences for each outcome with respect to app engagement using the categorized version of the number of user acts described earlier. Each outcome was evaluated separately for pre-PPA use and post-PPA use outcomes. Fisher exact tests were performed to test for differences across the 3 categories of user acts. Statistical significance was determined with a *P* value cutoff of .05.

## Results

### Demographic Differences Between PPA User and Nonuser Comparison Groups

Demographic characteristics between enrolled PPA users and the comparison cohort at the same clinic were compared. The unenrolled group were registered patients at the host clinic who either chose not to enroll or did not learn about the study. Table 1 provides the baseline characteristics and outcomes of the study sample groups. The young people with HIV studied across the PPA user and comparison groups were predominately male (310/373, 83.1%) and Black (257/373, 70.8%). At the start of the PPA study period, 69.2% (258/373) of patients had been out of HIV care. PPA participants, relative to the comparison group, were more likely to be younger, multiracial or *other* race, and newly diagnosed.

**Table 1:** Baseline characteristics of Positive Peers App (PPA) participants and nonparticipant comparison groups.

Characteristic		Total (n=373), n (%)	PPA (n=114), n (%)	Non-PPA (n=259), n (%)	*P* value^a^	
**Age group (years)**						<.001
	13-24	88 (23.6)	40 (35.1)	48 (18.5)		
	25-29	158 (42.4)	52 (45.6)	106 (40.9)		
	30-34	127 (34.1)	22 (19.3)	105 (40.5)		
**Sex at birth**						.12
	Male	310 (83.1)	100 (87.7)	210 (81.1)		
	Female	63 (16.9)	14 (12.3)	49 (18.9)		
**Race**						.006
	African American	257 (70.8)	78 (68.4)	179 (71.9)		
	White	83 (22.9)	22 (19.3)	61 (24.5)		
	Multiracial or other	23 (6.3)	14 (12.3)	9 (3.6)		
**Newly diagnosed**						<.001
	Yes	107 (28.7)	45 (39.5)	62 (23.9)		
	No, noncongenital	252 (67.6)	59 (51.8)	193 (74.5)		
	No, congenital	14 (3.8)	10 (8.8)	4 (1.5)		
**Out of care**						.008
	Yes	258 (69.2)	68 (59.7)	190 (73.4)		
	No	115 (30.8)	46 (40.4)	69 (26.6)		
**Office visits 6-12 months prior**						.91
	Yes	100 (27.2)	31 (27.2)	69 (26.6)		
	No	273 (72.8)	83 (72.8)	190 (73.4)		
**HIV laboratory test 6-12 months prior**						.88
	Yes	80 (21.5)	25 (21.9)	55 (21.2)		
	No	293 (78.6)	89 (78.1)	204 (78.8)		
**HIV viral suppression 6-12 months prior^b^**						.27
	Yes	61 (16.4)	15 (13.2)	46 (17.8)		
	No	312 (83.7)	99 (86.8)	213 (82.2)		

^a^*P* values generated from Pearson *χ*^2^ tests.

^b^HIV viral suppression defined as not detectable: <200 copies/mL.

### PPA Use Across Demographic Groups

Table 2 summarizes the types of PPA used across demographic groups. A total of 81.6% (93/373) of participants logged on to the PPA during the first 3 months following enrollment. The median number of log-ins by users was 9 (IQR 4.0-18.0), and the median number of user acts was 101 (IQR 46-183). Both median number of log-ins and user acts were lowest for the oldest 30 to 34 age group (vs other age groups), and the median number of user acts was higher for White users (vs African American), single (vs in a relationship), and “nonstraight” (vs “straight”) users. Although people employed full time logged into the app more than the other groups, unemployed participants showed the highest number of user acts. Similarly, the median number of log-ins was the highest for females, while the median number of user acts was the highest for males. Both median number of log-ins and user acts were highest for Latinx ethnicity (vs not Latinx), those newly diagnosed with HIV (vs not newly diagnosed), those carrying private or commercial insurance (vs other forms of insurance or no insurance), and those without a prior incarceration history (vs with an incarceration history).

**Table 2:** Positive Peers App use by patient characteristics (months 1-3).

Characteristic		Logged in? (n=114), n (%)			Number of times user logged in (n=93), median (IQR)		Number of user acts (n=93), median (IQR)
		Yes	No				
Overall		93 (81.6)	21 (18.4)	9.0 (4.0-18.0)		101.0 (46.0-183.0)	
**Age group (years)**							
	13-24	34 (85)	6 (15)	11.0 (4.0-18.0)		93.5 (48.0-161.0)	
	25-29	42 (80.8)	10 (19.2)	9.0 (5.0-18.0)		115.5 (65.0-183.0)	
	30-34	17 (77.3)	5 (22.7)	6.0 (3.0-21.0)		58.0 (30.0-214.0)	
**Sex at birth**							
	Male	82 (82)	18 (18)	8.5 (4.0-18.0)		104.0 (48.0-184.0)	
	Female	11 (78.6)	3 (21.4)	11.0 (2.0-19.0)		85.0 (30.0-151.0)	
**Race and ethnicity**							
	African American	61 (78.2)	17 (21.8)	8.0 (3.0-16.0)		94.0 (38.0-171.0)	
	White	19 (86.4)	3 (13.6)	9.0 (5.0-18.0)		140.0 (63.0-193.0)	
	Multiracial or other	13 (92.9)	1 (7.1)	9.0 (4.0-19.0)		88.0 (65.0-183.0)	
**Latinx**							
	Yes	12 (85.7)	2 (14.3)	12.5 (9.0-23.5)		145.5 (91.5-237.0)	
	No	81 (81)	19 (19)	8.0 (4.0-16.0)		93.0 (43.0-180.0)	
**Newly diagnosed**							
	Yes	38 (84.4)	7 (15.6)	12.0 (6.0-18.0)		118.5 (65.0-192.0)	
	No, noncongenital	46 (78)	13 (22)	6.5 (3.0-18.0)		97.5 (41.0-193.0)	
	No, congenital	9 (90)	1 (10)	9.0 (2.0-12.0)		65.0 (38.0-94.0)	
**Sexual preference**							
	Straight	21 (80.8)	5 (19.2)	8.0 (2.0-15.0)		70.0 (31.0-150.0)	
	Not straight	72 (81.8)	16 (18.2)	9.0 (5.0-18.0)		113.5 (57.0-188.0)	
**School completed**							
	HS^a^ graduate	75 (83.3)	15 (16.7)	9.0 (4.0-18.0)		102.0 (45.0-184.0)	
	Not an HS graduate	18 (75)	6 (25)	8.0 (3.0-18.0)		93.5 (46.0-169.0)	
**Employment status**							
	Full time	26 (92.9)	2 (7.1)	11.5 (5.0-18.0)		106.0 (70.0-193.0)	
	Part time	21 (84)	4 (16)	5.0 (4.0-16.0)		88.0 (42.0-182.0)	
	Unemployed	37 (75.5)	12 (24.5)	9.0 (5.0-18.0)		113.0 (45.0-183.0)	
	Other^b^	9 (75)	3 (25)	4.0 (2.0-12.0)		84.0 (38.0-150.0)	
**Health insurers**							
	No insurance	10 (90.9)	1 (9.1)	8.5 (4.0-16.0)		114.5 (48.0-161.0)	
	Medicaid or Medicare	60 (76)	19 (24)	8.0 (4.0-15.5)		84.5 (36.0-183.5)	
	Private	13 (100)	0 (0)	12.0 (8.0-22.0)		171.0 (106.0-248.0)	
	Other insurance	9 (100)	0 (0)	9.0 (5.0-21.0)		94.0 (88.0-137.0)	
**Relationship status**							
	Single and not dating anyone	64 (83.1)	13 (16.9)	9.0 (4.0-18.5)		104.0 (44.5-199.5)	
	In a relationship	29 (78.4)	8 (21.6)	8.0 (3.0-16.0)		94.0 (45.0-171.0)	
**Incarceration**							
	0 times	44 (84.6)	8 (15.4)	10.5 (4.5-18.0)		104.0 (42.5-214.5)	
	1-2 times	26 (83.9)	5 (16.1)	8.5 (5.0-14.0)		97.5 (58.0-169.0)	
	≥3 times	23 (74.2)	8 (25.8)	6.0 (3.0-18.0)		89.0 (31.0-310.0)	

^a^HS: high school.

^b^In school, disability.

### HIV Outcomes Between PPA Users and Nonusers

We were interested in determining whether there were significant differences across HIV outcomes within our cohort of PPA users. Table 3 presents the results of regressing 6- to 12-month postbaseline office visit attendance on PPA participation while adjusting for baseline characteristics and prebaseline medical records. The data suggest that no significant differences in clinic attendance exist based on the PPA participation status. In other words, the clinical comparison group was no different than the group of app users in prestudy period clinic attendance. After the app use period, there were no significant differences in PPA participation in HIV clinical outcomes based on race or new diagnosis or out-of-care status. However, among demographic variables, an interaction effect was detected for age. Age-stratified results suggested significantly improved outcomes for PPA users in the youngest (13-24 years) age group. Across the youngest age groups, PPA participants were more likely to obtain their HIV laboratory tests (adjusted odds ratio 2.85, 95% CI 1.03-7.90) than same-age nonparticipants. Importantly, younger PPA participants were also more likely to be virally suppressed (adjusted odds ratio 4.22, 95% CI 1.28-13.89) compared with nonparticipants. No significant differences in PPA participation in either HIV laboratory tests or viral suppression were observed among the older age groups.

**Table 3:** HIV outcomes between Positive Peers App (PPA) users and non-PPA comparison cohort.

		Outcomes^a^, aOR^b^ (95% CI)		
		Office visits	HIV laboratory tests	HIV viral suppression
**All patients^c^**				
	PPA	1.66 (0.99-2.80)	—^d^	—
	Non-PPA	Reference	—	—
**Age 13-24 years^e^**				
	PPA	—	2.85 (1.03-7.90)	4.22 (1.28-13.89)
	Non-PPA	—	Reference	Reference
**Age 25-29** **years**				
	PPA	—	1.86 (0.84-4.12)	1.07 (0.46-2.50)
	Non-PPA	—	Reference	Reference
**Age 30-34** **years**				
	PPA	—	0.54 (0.18-1.64)	0.45 (0.11-1.75)
	Non-PPA	—	Reference	Reference

^a^Outcomes are measured 6-12 months after baseline measure.

^b^aOR: adjusted odds ratio.

^c^Overall model adjusted for age, sex at birth, race, newly diagnosed, out of care, and 6-12 months prebaseline measures.

^d^When modeling outcomes, all-patient models are not relevant when age acts as an effect modifier, and age-stratified models are not relevant when age does not act as an effect modifier.

^e^Age-stratified models adjusted for sex at birth, race, newly diagnosed, out of care, and 6-12 months prebaseline measures.

### Effects of PPA Use on HIV Outcomes

Tables 4 and 5 illustrate that engagement in care and HIV viral suppression significantly improved following participation in the PPA (27.2% vs 52.6%, 21.9% vs 45.6%, and 13.2% vs 29.8%). Interestingly, before PPA enrollment, eventual high users of the app were less likely to have had office visits (7/43, 15%), to have a HIV laboratory test drawn (4/43, 9%), or to have been virally suppressed (2/43, 4%) than eventual nonusers or low to moderate users. As shown in the prestudy columns, before downloading the PPA, only 15% (7/43) of high users had office visits compared with 33.3% (7/114) and 36% (17/43) for eventual nonusers and low to moderate users, respectively. However, following PPA participation, these groups converged (11/21, 52%; 26/47, 55.3; vs 23/46, 50%), suggesting that app use facilitated engagement in care and viral suppression for patients most out of compliance.

**Table 4:** HIV outcomes (Office visits and HIV labs) before and after Positive Peers App (PPA) participation and stratified by app use (N=114).

Office visits?				Pre-PPA use		Post-PPA use		*P* value^a^		HIV laboratory tests?		Pre-PPA use		Post-PPA use		*P* value	
**Overall, n (%)**								<.001									<.001
	Yes		31 (27.2)		60 (52.6)				Yes		25 (21.9)		52 (45.6)				
	No		83 (72.8)		54 (47.4)				No		89 (78.1)		62 (54.4)				
**Number of user acts, n (%)**																	
	**None (n=21)**																
		Yes	7 (33.3)		11 (52.4)		—^b^		Yes		3 (14.3)		9 (42.9)		—		
		No	14 (66.7)		10 (47.6)		—		No		18 (85.7)		12 (57.1)		—		
	**Low-moderate (n=47)**																
		Yes	17 (36.2)		26 (55.3)		—		Yes		18 (38.3)		21 (44.7)		—		
		No	30 (63.8)		21 (44.7)		—		No		29 (61.7)		26 (55.3)		—		
	**High (n=46)**																
		Yes	7 (15.2)		23 (50.0)		—		Yes		4 (8.7)		22 (47.8)		—		
		No	39 (84.8)		23 (50.0)		—		No		42 (91.3)		24 (52.2)		—		
*P* value^c^				.05		.91		—		—		.002		.91		—	

^a^*P* values compare HIV outcomes before and after PPA participation using the McNemar test of agreement.

^b^Not applicable.

^c^*P* values compare the categories of user activity against Pre-PPA Post-PPA HIV outcomes using the Fisher exact test.

**Table 5:** HIV Viral suppression before and after Positive Peers App (PPA) participation and stratified by app use (N=114).

Viral suppression?						Pre-PPA use			Post-PPA use			*P* value^a^	
**Overall, n (%)**													<.001
	Yes				15 (13.2)			34 (29.8)			—^b^		
	No				99 (86.8)			80 (70.2)			—		
**Number of user acts, n (%)**													
	**None (n=21)**												
			Yes	2 (9.5)			5 (23.8)			—			
			No	19 (90.5)			16 (76.2)			—			
	**Low to moderate (n=47)**												
			Yes	11 (23.4)			16 (34.0)			—			
			No	36 (76.6)			31 (66.0)			—			
	**High (n=46)**												
			Yes	2 (4.4)			13 (28.3)			—			
			No	44 (95.6)			13 (71.7)			—			
*P* value^c^		—			.02			.72			—		

^a^*P* values compare HIV outcomes before and after PPA participation using the McNemar test of agreement.

^b^Not applicable.

^c^*P* values compare the categories of user activity against Pre-PPA Post-PPA HIV outcomes using the Fisher exact test.

## Discussion

### Principal Findings

This study aimed to assess the effects of PPA use on HIV clinical outcomes within a sample of out-of-care or newly diagnosed young people in one clinic community. Following at least 6 months of intervention participation, the youngest patient group (age 13-24 years) was more likely than the same-age nonuser cohort to see an improvement in completing laboratory tests and achieving or sustaining viral suppression. This is a significant finding because younger people are not only more likely to be unsuppressed and out of care but also burdened by psychosocial and behavioral risks that define this group as a high-impact target population for ending the HIV/AIDS epidemic [30,31].

It is notable that most PPA use occurred within the first 3 months of downloading the app. Although novelty may draw users to download and try an app, engagement quickly peaks as a function of continued exposure [32,33]. It may be that the knowledge gained from app engagement bolsters greater user health self-management. Specialized health apps such as PPA may facilitate positive habits such as tracking medication or marshaling support during difficult times. However, mobile app designers may also need to address novelty effects on study retention and design their recruitment and prospective data collection accordingly.

### Demographic Differences in Use

The most frequent PPA users were aged <30 years, newly diagnosed, and White or Latinx. Females logged into the app more often, but males engaged in more acts overall while using the app, possibly suggesting more surfing within the app or less purposeful use [34]. For example, females may prefer to use the app primarily to meet relational needs by using private chat or community forum functions. We caution that these demographic differences may be unique to a single-site location and should not be used for generalization to larger groups. However, these findings provide a reason to further explore these patterns across sites with a larger sample size. They could be markers of more complex behavioral patterns associated with mobile app uses and effects.

Among all PPA users, engagement in care and viral suppression improved 6 months from baseline, particularly for those in the highest app use category. Although we supported our prediction that greater involvement with the app would lead to greater effects, the mechanism underlying these effects remains obscured. Mere exposure to information is insufficient for explaining complex behavioral processes and outcomes [35]. Emerging models of user engagement with interactive digital media suggest that physical interaction with, and positive perceptions of, the technology interface predict greater cognitive involvement with provided content, which in turn motivates a user’s intent to manage, apply, and share that content [20,23,36]. Using this theoretical framework, we plan to conduct future research that will allow us to identify distinct use patterns over time to better understand how the PPA can be enhanced to support HIV self-management. We believe that freely available mobile apps such as the PPA could serve as a significant tool for managing the changing health and support needs as young people living with HIV adjust to a postdiagnosis life.

### Limitations

There are limitations associated with this work. Research volunteers may have been more motivated to maintain adherence, regardless of their enrollment in the mobile app. The app was assertively promoted in the clinic to all potential volunteers for 2 years. There are many reasons a patient may choose not to download a health app, including privacy concerns, data download costs, and past experience with apps. We had no quantitative data on people’s reasons for participating or declining participation. However, selection bias is a known risk in observational studies and may be evident if PPA users and nonusers were different in notable ways. Data from Table 1 suggest that nonusers skewed older and were more likely to be returning after a lapse in care rather than as a newly diagnosed patient. People returning to HIV care are experiencing a different affective or psychosocial experience than newly diagnosed younger adults. We need to determine whether these differences are sufficient to preclude these patients from considering using the PPA. In addition, it may be that young adults aged >25 years are more engaged in employment, established relationships, or other life responsibilities, reducing free time for app use. Finally, there were significant and unexpected differences in app use across races. Additional qualitative or mixed methods studies may reveal the nature of these differences and allow us to craft messaging and in-app tools tailored to their needs. Future analysis beyond this single clinic population will allow us to better determine additional interaction effects that may be contributing to this pattern.

Finally, a limitation is also found in the small pilot study sample size, limiting our ability to fully adjust data outcomes to additional relevant influences. The results are representative of our local public hospital community only. However, we are currently in the field collecting a larger and more diverse sample that will enable greater precision in our estimates. Along these lines, a randomized controlled trial design will present a more robust test of app impact. A key concern for future research is the determination of a potential threshold for app engagement to facilitate positive outcomes. Nevertheless, the results reported here confirm the usefulness of the PPA as a supportive tool for young people living with HIV. PPA engagement may be occurring at a particularly formative time following a new diagnosis or return to responsible care. In this way, the PPA is a feasible and effective patient-centered tool for facilitating engagement in care at a crucial turning point in HIV treatment.

### Implications

This study is predicated on the idea that greater engagement with a mobile app will facilitate the likelihood of achieving desirable HIV clinical outcomes. This idea of engagement or involvement in content is fundamental to modeling communication processes and effects [37]. Our findings support this hypothesis. Although the relationship between targeted digital content engagement and positive outcomes was supported, how that process occurs remains unseen. The cognitive or affective processes inherent in digital message processing are at the core of this process but not the sole determinant of a given outcome.

Current explanations of media use and effects are inclusive of the characteristics and features of various technologies to explain and ultimately predict outcomes [17,21,23]. Different technologies afford different experiences to users. Unlike general social media apps, the PPA affords users opportunities to tailor their anonymity as their confidence in the community grows, provides frequent social and medical information vetted by credible clinicians, and offers a supportive and monitored community that shares similar life experiences. Although there is variability across app users in terms of support needs and adjustment to living with HIV, these user differences can be intentionally targeted in app messaging and content [38,39]. This model frames our ongoing work and holds implications for future theorizing of mobile app uses and effects.

This study also has implications for HIV care. Table 1 shows the nonuser comparison cohort to include significantly more people returning to care after a lapse in the medical management of HIV. This returning-to-care population may face different psychosocial challenges as they resume an adherent lifestyle. Continued targeting of this group may address a noted literature gap regarding best practices for re-engagement in care [40-42].

Importantly, the youngest set of PPA users was most likely to realize positive outcomes 3 months after enrollment. This population of people with HIV is of greatest concern to ending the epidemic efforts. Data presented here suggest that the app had a positive impact on these groups. Counselors and clinics who care for people living with HIV need socially relevant tools for younger patients. This is particularly relevant for rural communities or young people with HIV who desire remote, around-the-clock community support. Within these communities, the PPA offers acceptance, tangible support, self-management tools, and credible HIV-relevant information in one place.

### Conclusions

HIV self-management is a significant challenge for young people that can be alleviated with the use of mobile apps that bring health information, tools, and supports directly to wherever they may be. Given that new HIV cases are predominantly among younger people, this approach is crucial for achieving an undetectable HIV status for young people living with HIV. Furthermore, acceptance into a knowing and supportive community may be a key resource for increasing HIV literacy and lessening internalized stigma [42]. The PPA is currently available via Google and iOS app stores, although users are required to verify their age and diagnosis via an electronic onboarding system. These data, taken with previously published results, point to the PPA as a useful tool for helping young people living with HIV achieve clinical outcomes that will both preserve their health and contribute to ending the epidemic.

## Abbreviations

PPA: Positive Peers App
